# The Neuroprotective Effects of SIRT1 on NMDA-Induced Excitotoxicity

**DOI:** 10.1155/2017/2823454

**Published:** 2017-09-01

**Authors:** Xiaorong Yang, Peipei Si, Huaping Qin, Litian Yin, Liang-Jun Yan, Ce Zhang

**Affiliations:** ^1^National Key Disciplines, Key Laboratory for Cellular Physiology of Ministry of Education, Department of Neurobiology, Shanxi Medical University, No. 56 Xin Jian South Road, Taiyuan, 030001 Shanxi, China; ^2^Key Laboratory of Neurology of Hebei Province, The Second Hospital of Hebei Medical University, Shijiazhuang, 050071 Hebei, China; ^3^Department of Pharmaceutical Sciences, UNT System College of Pharmacy, University of North Texas Health Science Center, Fort Worth, TX 76107, USA

## Abstract

Silent information regulator 1 (SIRT1), an NAD^+^-dependent deacetylase, is involved in the regulation of gene transcription, energy metabolism, and cellular aging and has become an important therapeutic target across a range of diseases. Recent research has demonstrated that SIRT1 possesses neuroprotective effects; however, it is unknown whether it protects neurons from NMDA-mediated neurotoxicity. In the present study, by activation of SIRT1 using resveratrol (RSV) in cultured cortical neurons or by overexpression of SIRT1 in SH-SY5Y cell, we aimed to evaluate the roles of SIRT1 in NMDA-induced excitotoxicity. Our results showed that RSV or overexpression of SIRT1 elicited inhibitory effects on NMDA-induced excitotoxicity including a decrease in cell viability, an increase in lactate dehydrogenase (LDH) release, and a decrease in the number of living cells as measured by CCK-8 assay, LDH test, and Calcein-AM and PI double staining. RSV or overexpression of SIRT1 significantly improved SIRT1 deacetylase activity in the excitotoxicity model. Further study suggests that overexpression of SIRT1 partly suppressed an NMDA-induced increase in p53 acetylation. These results indicate that SIRT1 activation by either RSV or overexpression of SIRT1 can exert neuroprotective effects partly by inhibiting p53 acetylation in NMDA-induced neurotoxicity.

## 1. Introduction

Silent information regulator 1 (SIRT1), an NAD^+^-dependent deacetylase, is known to deacetylate histone and nonhistone proteins such as transcription factors. It participates in a variety of physiopathological processes such as health maintenance in development, gametogenesis, homeostasis, longevity, and several neurodegenerative diseases as well as age-related disorders [1–5]. Recently, the neuroprotective effects of SIRT1 have attracted great interest. It has been found that SIRT1 could be upregulated to antagonize neuronal injury in different animal models, such as cerebral ischemia, Alzheimer's disease (AD), and Huntington's disease (HD) [6]. It has also been demonstrated that SIRT1 deacetylates p53, PGC-1*α*, and NF-*κ*B to prevent many pathogenic processes. However, it remains unknown whether SIRT1 protects neurons from NMDA-mediated neurotoxicity in different excitotoxic insult models.

Glutamate is a primary excitatory amino acid neurotransmitter and activation of glutamate receptors including NMDA receptor plays crucial roles in the central nervous system. However, overactivation of NMDA receptor may cause intracellular calcium overload, leading to an enzymatic cascade of events resulting ultimately in cell death known as excitotoxicity [7]. A wide range of acute and chronic brain injury diseases, such as stroke/ischemia and epilepsy, and certain neurodegenerative disorders have been linked to NMDA receptor-mediated excitotoxicity [8]. Therefore, NMDA-induced excitotoxicity is a useful tool to evaluate neurotoxicity in isolated cells and is a good model of nerve injury that mimics closely the situation *in vivo* [9].

The present study was designed to investigate the neuroprotection of SIRT1 in NMDA-induced excitotoxicity by activation of SIRT1 using resveratrol (RSV) in cultured cortical neurons or by overexpression of SIRT1 in the SH-SY5Y cell line. The neuroprotective role of SIRT1 activity *in vitro* may be useful for the development of new treatments for central nervous system disorders.

## 2. Experimental Procedures

### 2.1. Reagents

Neurobasal/B27, DMEM/F-12, and fetal bovine serum (FBS) were purchased from Gibco-BRL (Grand Island, NY, USA). Lipofectamine 2000 transfection reagents were obtained from life technologies (St. Louis, MO, USA). Poly-D-lysine (MW 150,000–300,000), trypsin, arabinoside cytosine, Calcein-AM, propidium iodide (PI), RSV, Sirtinol, NMDA, MK-801, and SIRT1 assay kit were all purchased from Sigma-Aldrich (St. Louis, MO, USA). The Cell Counting Kit-8 (CCK-8) was from Dojindo, and the kit of LDH was from Njjcbio. The polyclonal antibody to SIRT1 was from Santa Cruz Biotechnology (Santa Cruz, CA, USA). Two polyclonal antibodies to p53 and Ace-p53 were obtained from Cell Signaling Technology (Beverly, MA, USA).

### 2.2. Cell Culture

Primary cortical cells were isolated from 1–3-day-old Wistar rats and were cultured as previously described [10]. In brief, cortical neurons from rats anesthetized with ketamine (intraperitoneal injection, 100 mg/kg, 3 min) were dissected and digested in 0.025% trypsin, followed by centrifugation at 800 g for 5 min. Cells were resuspended in neurobasal/B27 medium and cultured at 37°C in 5% CO_2_. Arabinoside cytosine (10 *μ*M) was added after 24 h *in vitro* to inhibit non-neuronal cell growth. Experiments were performed after 10–12 days *in vitro*.

The human neuroblastoma SH-SY5Y cell, obtained from the Chinese Academy of Sciences Institute of Cell Resource Center, Shanghai, China, was maintained under a DMEM/F12 medium with 10% FBS in 5% CO_2_ incubator. They were washed by PBS buffer before adding 0.25% Trypsin-EDTA, followed by incubation for 5 min at room temperature. Then, the cells were detached, resuspended in medium, counted, and seeded into plates at the density of 1 × 10^5^.

### 2.3. NMDA Treatment

After overnight incubation allowing the cells to reach 80% confluency, cells were treated with NMDA-containing Mg^2+^-free Locke's buffer for 2 h. RSV was added to cultures 12 h prior to NMDA induction. Sirtinol was added 2 h before NMDA treatment. MK-801 and NMDA was simultaneously added to Mg^2+^-free Locke's buffer in the NMDA + MK-801 group. Control cells were incubated with drug-free Mg^2+^-free Locke's buffer and grown at 37°C in an atmosphere containing 5% CO_2_.

### 2.4. Transfection of SIRT1

The expression vector expressing human wild-type SIRT1 (WT-SIRT1) and the dominant-negative form of human SIRT1 (DN-SIRT1) was constructed by Genecopoeia. The plasmids were extracted with a Plasmid Midi Kit (Omega, GA, USA). The SH-SY5Y cells were seeded into plates at a density of 1 × 10^5^, and after 24 h, the plasmids were transfected into the cells with a Lipofectamine 2000 Transfection Reagent.

### 2.5. Cell Viability Assay

Cells were seeded in 96-well plates, and cell viability was assayed 24 h after NMDA exposure. Administration of 10 *μ*L cck-8 solution into each well was performed followed by incubation at 37°C for 2 h. Absorbance at 490 nm was measured using a microplate reader (Packard, Meridien, MS).

### 2.6. Lactate Dehydrogenase (LDH) Assay

LDH is released from cells into a culture medium upon cell lysis. The cells were plated in 24-well plates. At 24 h after NMDA exposure, the supernatant was collected to measure LDH release according to the manufacturer's instructions.

### 2.7. Calcein-AM and PI Staining

Calcein-AM solution (20 *μ*M) was added to coverslips and the cells were incubated at 37°C for 30 min. PI solution was added and the cells were incubated at 37°C for 5 min. The cells were examined by using confocal microscope (Olympus, FV-1000) at the excitation wave of 490 nm and emission wave of 515 nm.

### 2.8. SIRT1 Deacetylase Activity Assay

To measure SIRT1 activity, the protein was extracted from cells. The enzyme activity of SIRT1 was measured using a SIRT1 assay kit (CS1040; Sigma-Aldrich) based on the fleur de Lys-SIRT1 substrate peptide. The fluorescence intensity was measured with a microplate reader (Packard, Meridien, MS), and the excitation wavelength was 365 nm, and the emission wavelength was 460 nm.

### 2.9. Quantitative Real-Time PCR (qRT-PCR)

Total RNA from SH-SY5Y cell was isolated using TRIzol reagent (Invitrogen Life Technologies, Carlsbad, CA, USA) according to the manufacturer's instruction. Reverse transcription was performed with High-Capacity cDNA Archive Kit (Applied Biosystem). qRT-PCR primers were synthesized by the software of Primer Premier according to the following sequences: *β*-actin (forward primer: 5′-TCATCACCATTGGCAATGAG-3′, reverse primer: 5′-CACTGTGTTGGCGTACAGGT-3′); SIRT1: (forward primer: 5′-GCCTCACATGCAAGCTCTAGTGACT-3′, reverse primer: 5′-ACTCAGGTGGAG GTATTGTTTCCG-3′). qRT-PCR was performed using StepOne Real-Time PCR Detection System (ABI) and SYBR premix EX taqII (Takara).

### 2.10. Western Blot Analysis

The SH-SY5Y cells were collected at 24 h after exposure to NMDA. Then, cells were lysed in a lysis buffer (10 mM Tris-HCl (pH 7.4), 1 mM EDTA, and 1% Triton X-100). Cleared cell lysates were obtained after centrifugation at 10000 ×g for 30 min at 4°C. After measurement of protein concentration using a BCA Protein Assay kit, cell lysates (30 ~ 50 *μ*g/lane) were subjected to SDS-PAGE, and separated proteins were electrotransferred to nitrocellulose membranes. The membranes were washed in Tris-buffered saline (TBS) containing 0.1% Tween 20 and 3% bovine serum albumin (BSA). The membranes were incubated overnight at 4°C in TBS containing 3% BSA and one of the following primary antibodies: SIRT1 (1 : 100), p53 (1 : 1000), and Ace-p53 (1 : 1000). Subsequently, the labeled proteins were incubated with an HRP-conjugated anti-rabbit IgG (1 : 10,000) for 2 h. Blots were developed with the ECL chemiluminescence system and were captured on autoradiographic films (Kodak Image Station 440). Films were scanned and a densitometric analysis of the bands was performed with AlphaEase image analysis software.

### 2.11. Statistical Analysis

The data were expressed as means ± S.E.M. of at least three independent experiments. One-way analysis of variance (ANOVA) with Bonferroni post hoc test was used for statistical comparisons. *P* < 0.05 was considered to be significant.

## 3. Results

### 3.1. Neuroprotective Effects of RSV on NMDA-Induced Excitotoxicity in Primary Neurons

#### 3.1.1. Effects of RSV on NMDA-Induced Decrease in Cell Viability

**Figure 1:**
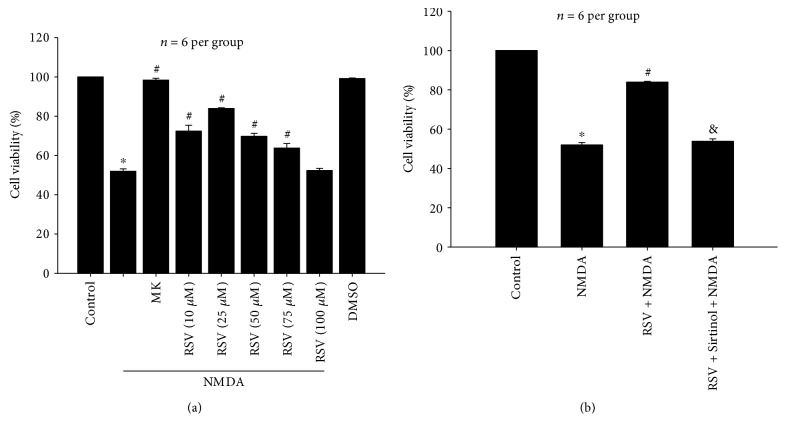
Effects of RSV on NMDA-induced decrease in cell viability in primary neurons. (a) Pretreatment of RSV (10 *μ*M, 25 *μ*M, 50 *μ*M, and 75 *μ*M) improved cell viability compared with the NMDA treatment group (*P* < 0.05). (b) RSV (25 *μ*M) significantly reversed NMDA-induced decrease in cell viability (*P* < 0.05), and Sirtinol (10 *μ*M) inhibited the effect of RSV (*P* < 0.05). RSV: resveratrol; MK: MK-801. Each value represents the mean ± S.E.M. of six independent experiments. ^∗^*P* < 0.05 versus the control group, ^#^*P* < 0.05 versus the NMDA group, ^&^*P* < 0.05 versus the RSV + NMDA group.

Our previous study showed that the optimal excitotoxicity was induced 24 h after NMDA (100 *μ*M) exposure for 2 h in primary cortical neurons.  Figure 1(a) shows that NMDA-induced cell viability decreased by 51.97% as compared to the control in primary neurons (*P* < 0.05). Pretreatment with five dosages (10 *μ*M, 25 *μ*M, 50 *μ*M, 75 *μ*M, and 100 *μ*M) of RSV, a potent SIRT1 activator, showed that cell viability was increased by 20.43% (*P* < 0.05), 31.92% (*P* < 0.05), 17.78% (*P* < 0.05), 11.85% (*P* < 0.05), and 0.37% (*P* > 0.05), respectively, when compared to that of the NMDA-treated group (Figure 1(a)). In the following experiments, RSV was administered at the concentration of 25 *μ*M based on the significant protective effect observed in the 25 *μ*M RSV group. MK-801 (10 *μ*M) did not alter cell viability and yielded a value nearly equivalent to that of the control group (*P* > 0.05). These data support the notion that the toxic effects were induced by NMDA. Similarly, DMSO as a NMDA vehicle had no significant effects on cell viability (*P* > 0.05).

As shown in Figure 1(b), pretreatment with RSV (25 *μ*M) significantly increased the viability of primary neurons compared to that of the NMDA group (*P* < 0.05). However, a combination of Sirtinol (10 *μ*M) and RSV (25 *μ*M) did not affect the NMDA-induced decrease in cell viability (*P* > 0.05), suggesting that Sirtinol, a specific inhibitor of SIRT1, blocked the protective effect of RSV on NMDA-induced excitotoxicity. Pretreatment of Sirtinol (10 *μ*M) alone does not affect cell survival in primary neurons (data not shown).

#### 3.1.2. Effects of RSV on NMDA-Induced LDH Release

**Figure 2:**
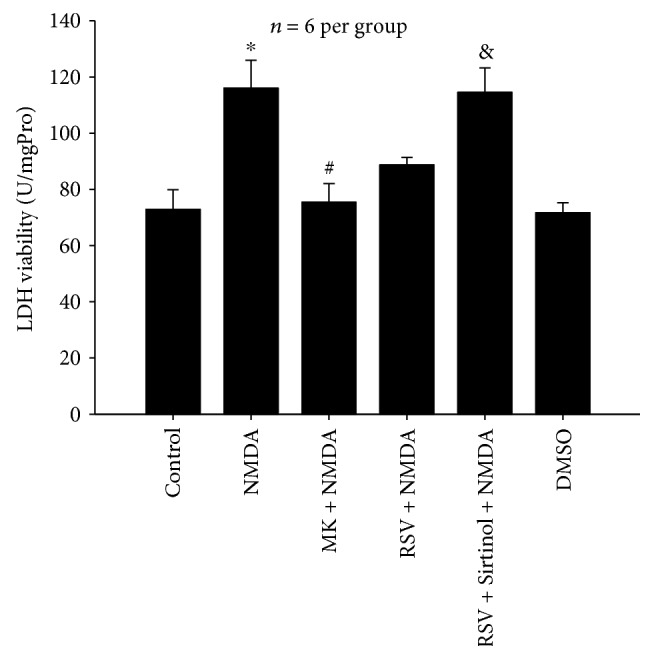
Effects of RSV on NMDA-induced LDH release in primary neurons. RSV (25 *μ*M) reduced NMDA-induced LDH release (*P* < 0.05), and Sirtinol (10 *μ*M) abolished the role of RSV (*P* < 0.05). RSV: resveratrol; MK: MK-801. Each value represents the mean ± S.E.M. of six independent experiments. ^∗^*P* < 0.05 versus the control group, ^#^*P* < 0.05 versus the NMDA group. ^&^*P* < 0.05 versus the RSV + NMDA group.

After treatment of NMDA, LDH levels rose by 116.03% compared with those of the control group (*P* < 0.05, Figure 2). NMDA-induced LDH release was fully blocked by MK-801 (10 *μ*M). Administration of RSV (25 *μ*M) reduced NMDA-induced LDH release by 27.34% (*P* < 0.05), and pretreatment with Sirtinol (10 *μ*M) abolished the role of RSV (*P* < 0.05). There was no difference in LDH release between the DMSO group and the control group (*P* > 0.05).

#### 3.1.3. Effects of RSV on NMDA-Induced Decrease in the Number of Living Cells

**Figure 3:**
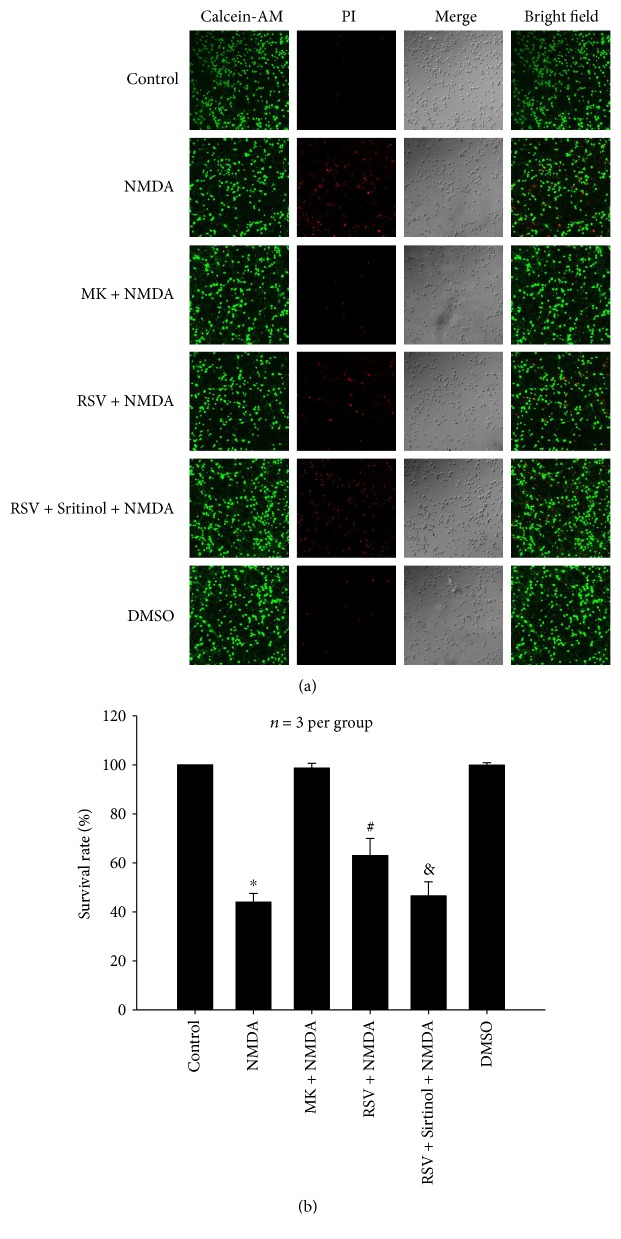
Effects of RSV on NMDA-induced decrease in the number of living cells in primary neurons. (a) Representative micrographs showing the suppression of RSV (25 *μ*M) on NMDA-induced decrease of living cells (*P* < 0.05), which was abolished by Sirtinol (10 *μ*M) (*P* < 0.05). Living cells were stained by Calcein-AM (green), and dead cells were stained by PI (red). (b) Bar graph of mean of living cells. RSV: resveratrol; MK: MK-801. Each value represents the mean ± S.E.M. of three independent experiments. ^∗^*P* < 0.05 versus the control group, ^#^*P* < 0.05 versus the NMDA group. ^&^*P* < 0.05 versus the RSV + NMDA group.

Exposure to NMDA resulted in a significant decrease in the cell survival rate estimated by Calcein-AM and PI staining (Figure 3). After treatment with NMDA, the cell survival rate decreased by 55.98% as compared to that of the control group (*P* < 0.05). MK-801 (10 *μ*M) completely inhibited NMDA-induced decrease of living cells. Administration of RSV (25 *μ*M) significantly increased the cell survival rate by 18.99% as compared to the NMDA-treated group (*P* < 0.05) and pretreatment with Sirtinol (10 *μ*M) abolished the role of RSV (*P* < 0.05).

#### 3.1.4. Effects of RSV on NMDA-Induced Decrease in SIRT1 Deacetylase Activity

**Figure 4:**
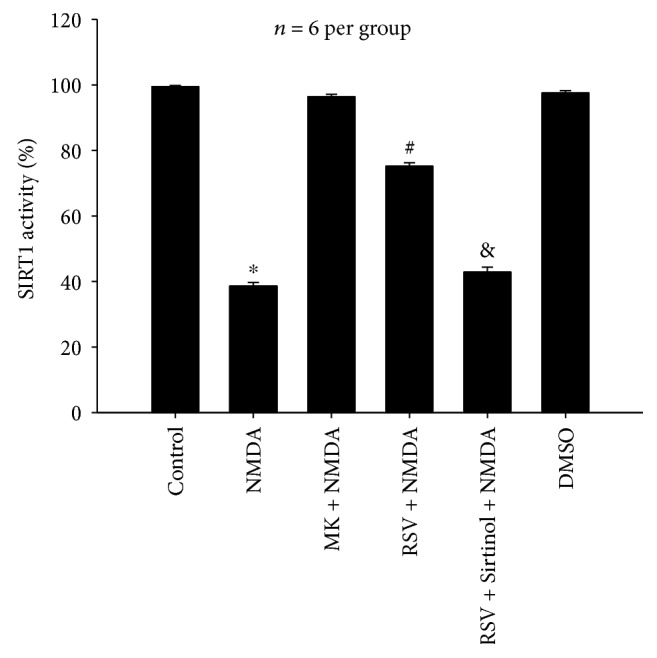
Effects of RSV on NMDA-induced decrease of SIRT1 deacetylase activity in primary neurons. RSV (25 *μ*M) significantly ameliorated SIRT1 activity reduced by NMDA (*P* < 0.05), and Sirtinol (10 *μ*M) abolished the effect of RSV (*P* < 0.05). MK: MK-801. Each value represents the mean ± S.E.M. of six independent experiments. ^∗^*P* < 0.05 versus the control group, ^#^*P* < 0.05 versus the NMDA group, ^&^*P* < 0.05 versus the RSV + NMDA group.

As shown in Figure 4, NMDA greatly reduced the SIRT1 activity (*P* < 0.05), which was inhibited by MK-801. Pretreatment with RSV significantly ameliorated SIRT1 activity reduced by NMDA (*P* < 0.05), while Sirtinol abolished the effect of RSV (*P* < 0.05). There was no difference in SIRT1 deacetylase activity between the DMSO group and the control group (*P* > 0.05).

### 3.2. Neuroprotective Effects of SIRT1 on NMDA-Induced Excitotoxicity in the SH-SY5Y Cell Line

#### 3.2.1. NMDA-Induced Decrease in Cell Viability

**Figure 5:**
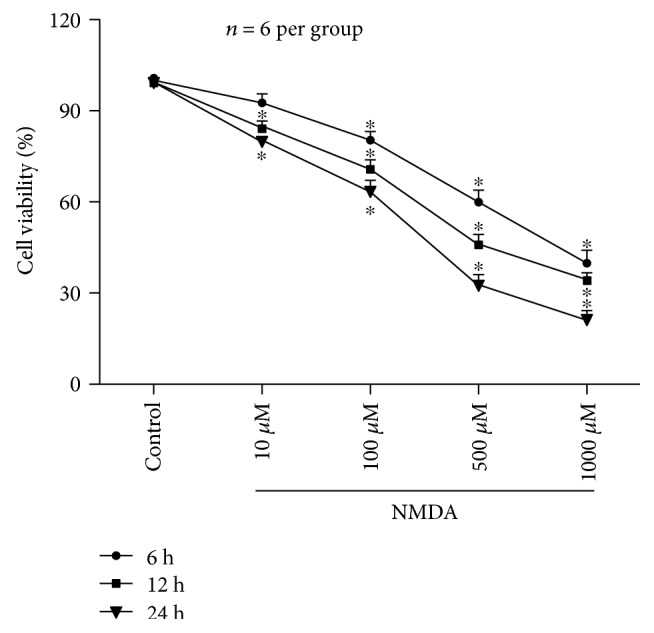
Effects of different concentrations (10–1000 *μ*M) of NMDA on cell viability in SH-SY5Y cell. NMDA (100 *μ*M, 500 *μ*M, and 1000 *μ*M) decreased cell viability at 6 h, 12 h, and 24 h after NMDA exposure for 2 h (*P* < 0.05). Only at 12 h and 24 h after NMDA (10 *μ*M), treatment was significant (*P* < 0.05). Numbers represent the percentage of the living cells normalized to the control. Each value represents the mean ± S.E.M. of six independent experiments. ^∗^*P* < 0.05 versus the control group.

To better characterize NMDA-induced neuronal insults of the SH-SY5Y cell line, the administration of NMDA at different concentrations (10 *μ*M, 100 *μ*M, 500 *μ*M, and 1000 *μ*M) and the cell viability were measured 6 h, 12 h, and 24 h after NMDA exposure for 2 h. Results showed that 500 *μ*M NMDA decreased cell viability by 49.26% (*P* < 0.05) 12 h after NMDA exposure (Figure 5), which were used as an insult-induced model for further experiments. In SH-SY5Y cell, NMDA decreased cell viability, which could be antagonized by MK-801 (data not shown).

#### 3.2.2. Overexpression of SIRT1 Increased the Levels of SIRT1 mRNA and Protein in NMDA-Induced Excitotoxicity

**Figure 6:**
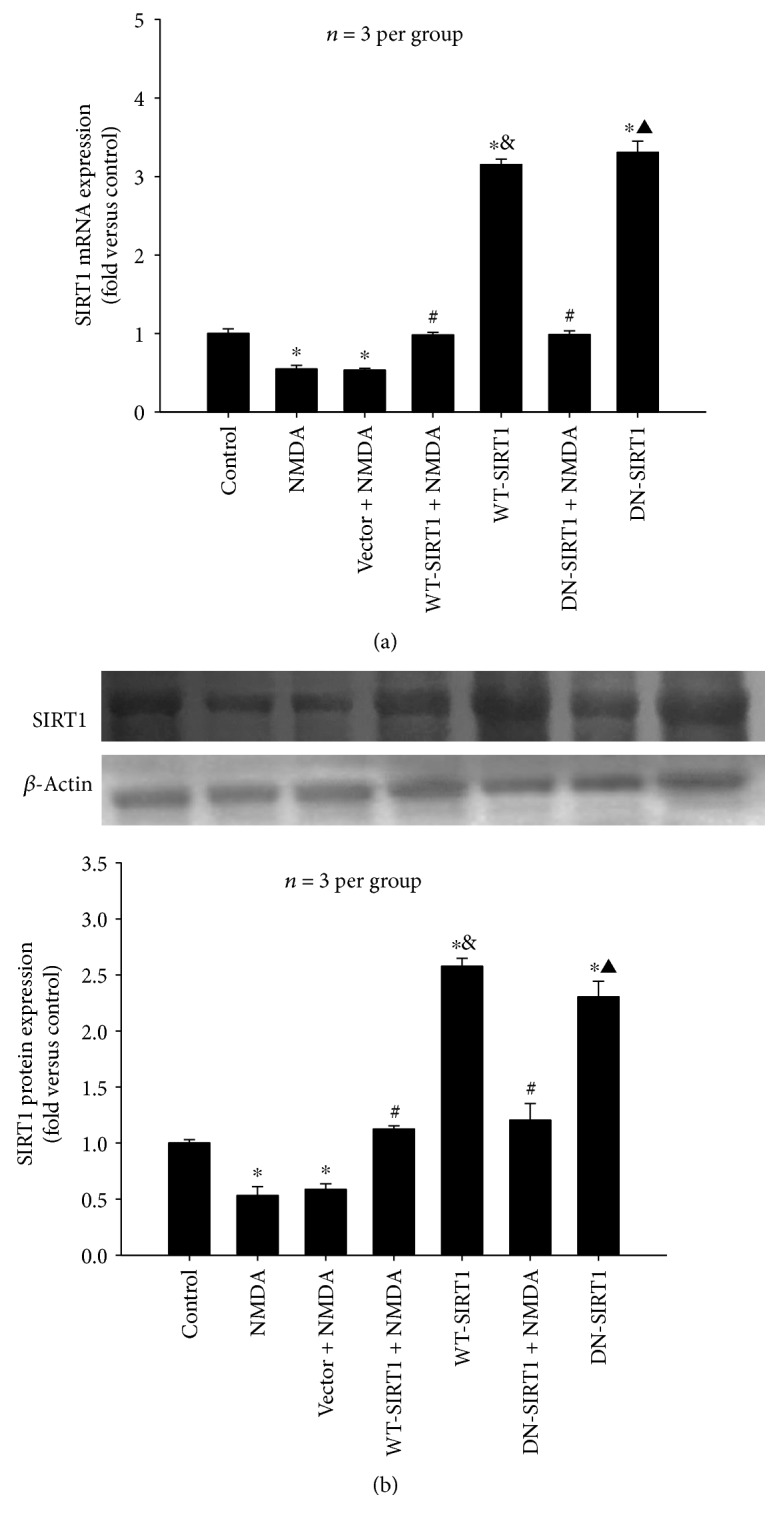
Overexpression of SIRT1 (WT-SIRT1 or DN-SIRT1) increased the levels of SIRT1 mRNA and protein reduced by NMDA (500 *μ*M) in SH-SY5Y cell (*P* < 0.05). (a) Quantitative representations of SIRT1 mRNA by bar graph. (b) Western blot probed with antibodies against SIRT1 and *β*-actin (upper panel) and quantitative representations of SIRT1 protein expression by bar graph (lower panel). Each value represents the mean ± S.E.M. of three independent experiments. ^∗^*P* < 0.05 versus the control group. ^#^*P* < 0.05 versus the vector + NMDA group, ^&^*P* < 0.05 versus the WT-SIRT1 + NMDA group, ^▲^*P* < 0.05 versus the DN-SIRT1 + NMDA group.

NMDA significantly decreased the level of SIRT1 mRNA (Figure 6(a)) and SIRT1 protein (Figure 6(b)) when compared with that of the control group (*P* < 0.05). WT-SIRT1/DN-SIRT1 overexpression restored the NMDA-induced decrease in the levels of SIRT1 mRNA and protein compared to those of the control (*P* < 0.05); however, WT-SIRT1/DN-SIRT1-overexpressing cells without NMDA treatment exhibited about a 2 ~ 3-fold increase in SIRT1 mRNA and protein when compared with those of the control group (*P* < 0.05). The level of SIRT1 mRNA and protein showed a great difference between the WT-SIRT1 + NMDA group and the WT-SIRT1 group and also between the DN-SIRT1 + NMDA and DN-SIRT1 groups (*P* < 0.05), indicating that the transfection efficiency may be downregulated by NMDA. There was no difference between the NMDA group and the NMDA + vector group (*P* > 0.05).

#### 3.2.3. Effects of SIRT1 Overexpression on the Deacetylase Activity in NMDA-Induced Excitotoxicity

**Figure 7:**
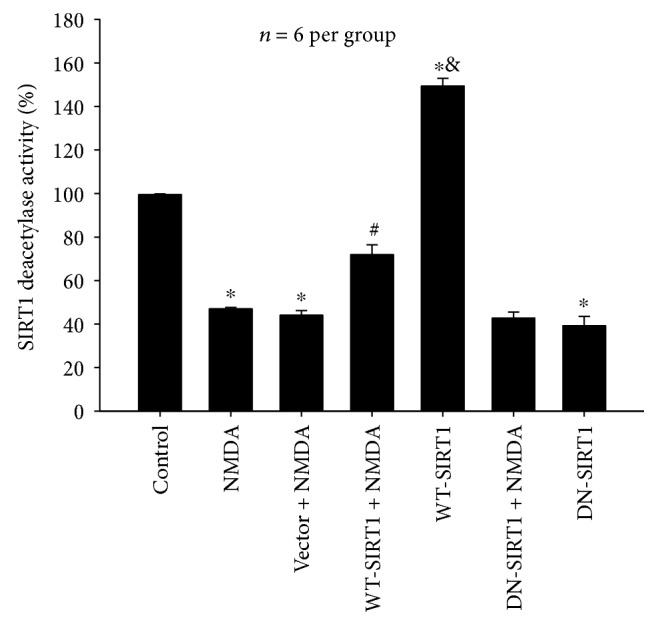
Effects of SIRT1 overexpression on the deacetylase activity in NMDA-induced excitotoxicity of SH-SY5Y cell. WT-SIRT1 overexpression significantly reversed the deacetylase activity decreased by NMDA (*P* < 0.05), and DN-SIRT1 overexpression had no effect (*P* > 0.05). Each value represents the mean ± S.E.M. of six independent experiments. ^∗^*P* < 0.05 versus the control group. ^#^*P* < 0.05 versus the vector + NMDA group, ^&^*P* < 0.05 versus the WT-SIRT1 + NMDA group.

As shown in Figure 7, NMDA inhibited SIRT1 deacetylase activity (*P* < 0.05). WT-SIRT1 overexpression after exposure to NMDA reversed the deacetylase activity decreased by NMDA (*P* < 0.05), whereas DN-SIRT1 overexpression with NMDA administration had no effect (*P* > 0.05). Compared with that of the control group, WT-SIRT1 overexpression itself significantly increased SIRT1 activity (*P* < 0.05), and DN-SIRT1 overexpression itself reduced the activity (*P* < 0.05). The SIRT1 activity showed a great difference between the WT-SIRT1 + NMDA group and the WT-SIRT1 group (*P* < 0.05). There was no difference between the DN-SIRT1 + NMDA and DN-SIRT1 group and also between the NMDA group and the NMDA + vector group (*P* > 0.05).

#### 3.2.4. Effects of SIRT1 Overexpression on p53 Acetylation in NMDA-Induced Excitotoxicity

**Figure 8:**
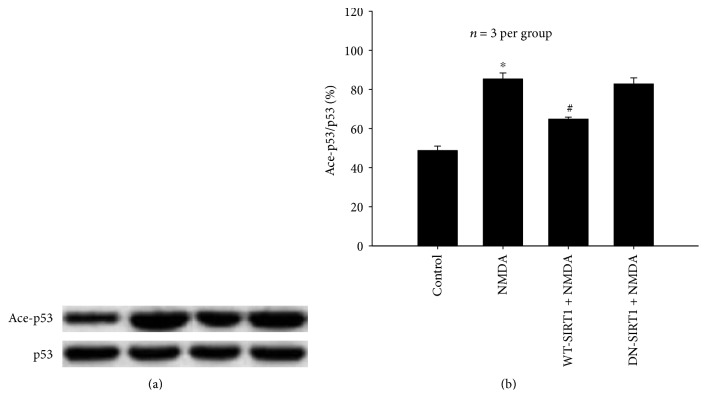
Effects of SIRT1 overexpression on p53 acetylation in NMDA-induced excitotoxicity of SH-SY5Y cell. WT-SIRT1 overexpression partially inhibited NMDA-stimulated p53 acetylation (*P* < 0.05), and DN-SIRT1 overexpression had no effect (*P* > 0.05). (a) Western blot probed with antibodies against p53 and Ace-p53. (b) Quantitative representations of Ace-p53 by bar graph. Each value represents the mean ± S.E.M. of three independent experiments. ^∗^*P* < 0.05 versus the control group, ^#^*P* < 0.05 versus the NMDA group.

Figure 8 shows that NMDA induced acetylation of p53 and the level of acetylated p53 (Ace-p53) was significantly higher (36.60%) than that of the control group (*P* < 0.05). WT-SIRT1 overexpression partially inhibited NMDA-stimulated p53 acetylation (*P* < 0.05), and DN-SIRT1 overexpression had no effect on Ace-p53 increased by NMDA (*P* > 0.05). The total levels of p53 were virtually unchanged under all of these experimental conditions.

#### 3.2.5. Effects of SIRT1 Overexpression on the Cell Viability Reduced by NMDA

**Figure 9:**
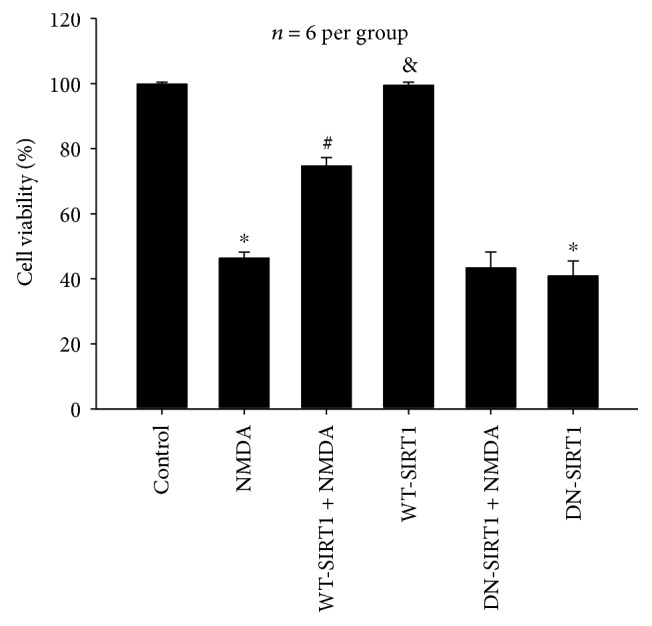
Effects of SIRT1 overexpression on cell viability reduced by NMDA in the SH-SY5Y cell line. WT-SIRT1 overexpression reversed NMDA-induced decrease in cell viability (*P* < 0.05), and DN-SIRT1 overexpression did not affect cell viability in the NMDA group (*P* > 0.05). Each value represents the mean ± S.E.M. of six independent experiments. ^∗^*P* < 0.05 versus the control group, ^#^*P* < 0.05 versus the NMDA group, ^&^*P* < 0.05 versus the WT-SIRT1 + NMDA group.

Figure 9 shows that WT-SIRT1 overexpression reversed NMDA-induced decrease in cell viability (*P* < 0.05), while DN-SIRT1 overexpression did not affect cell viability in the NMDA group (*P* > 0.05). WT-SIRT1 overexpression alone did not affect cell viability (*P* > 0.05), whereas DN-SIRT1 overexpression alone reduced cell viability (*P* < 0.05). There was a difference between the WT-SIRT1 + NMDA and WT-SIRT1 groups (*P* < 0.05); however, there was no difference between the DN-SIRT1 + NMDA and DN-SIRT1 groups (*P* > 0.05).

#### 3.2.6. Effects of SIRT1 Overexpression on NMDA-Induced LDH Release

**Figure 10:**
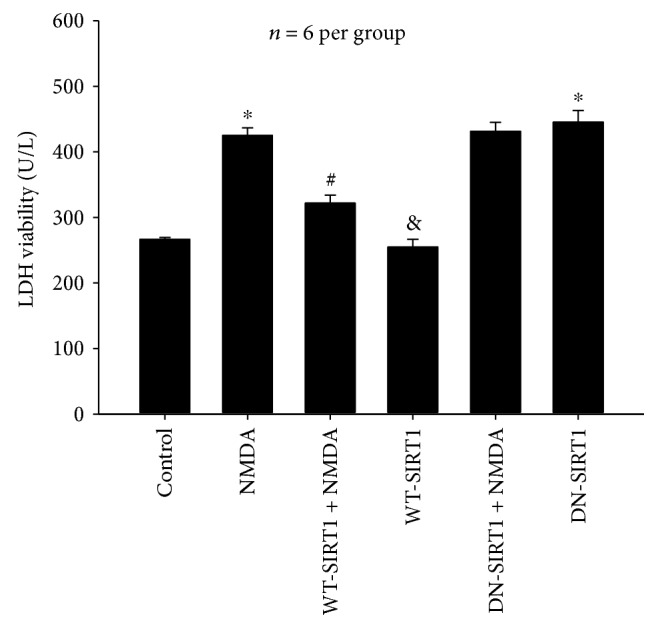
Effects of SIRT1 overexpression on NMDA-induced the amount of LDH release the in SH-SY5Y cell line. WT-SIRT1 overexpression reduced NMDA-induced LDH release (*P* < 0.05), and DN-SIRT1 overexpression did not protect against NMDA-mediated LDH release (*P* > 0.05). Each value represents the mean ± S.E.M. of six independent experiments. ^∗^*P* < 0.05 versus the control group, ^#^*P* < 0.05 versus the NMDA group, ^&^*P* < 0.05 versus the WT-SIRT1 + NMDA group.

As shown in Figure 10, WT-SIRT1 overexpression reduced NMDA-induced LDH release by 24.26% (*P* < 0.05). Whereas, DN-SIRT1 overexpression did not protect against NMDA-mediated LDH release (*P* > 0.05). The effects of WT-SIRT1 or DN-SIRT1 overexpression alone on LDH release were completely consistent with those of WT-SIRT1 or DN-SIRT1 overexpression alone on cell viability.

#### 3.2.7. Effects of SIRT1 Overexpression on the Number of Living Cells Reduced by NMDA

**Figure 11:**
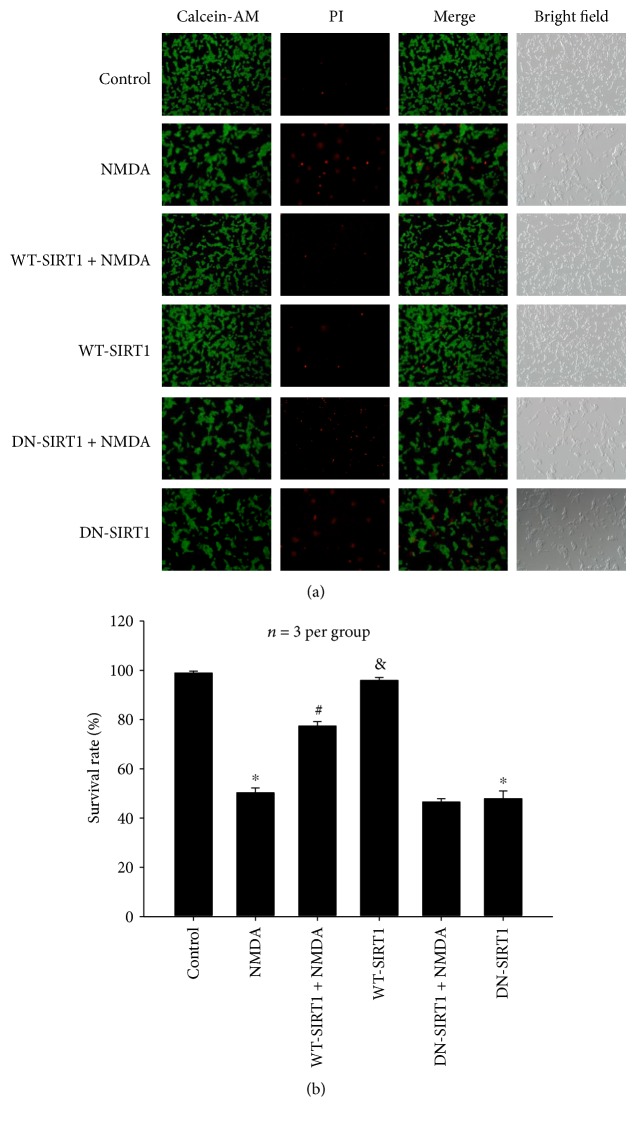
Effects of SIRT1 overexpression on the number of living cells reduced by NMDA in the SH-SY5Y cell line. (a) Representative micrographs showing the suppression of WT-SIRT1 overexpression on NMDA-induced decrease of living cells (*P* < 0.05) and no effect of DN-SIRT1 overexpression on the number of survival cells in the NMDA group (*P* > 0.05). Living cells were stained by Calcein-AM (green), and dead cells were stained by PI (red). (b) Bar graph of mean of living cells. Each value represents the mean ± S.E.M. of three independent experiments. ^∗^*P* < 0.05 versus the control group, ^#^*P* < 0.05 versus the NMDA group, ^&^*P* < 0.05 versus the WT-SIRT1 + NMDA group.

Calcein-AM and PI staining results (Figure 11) showed that NMDA resulted in a significant decrease in the number of living cells, which was inhibited by WT-SIRT1 overexpression (*P* < 0.05). While DN-SIRT1 overexpression has no effect on the number of survival cells when compared with the NMDA group (*P* > 0.05). The effects of WT-SIRT1 or DN-SIRT1 overexpression alone on cell survival showed similar results as those of the data described above.

## 4. Discussion

The present study provided the following three important findings. First, activation of SIRT1 or overexpression of SIRT1 protected against NMDA-mediated excitotoxicity; second, the neuroprotective effects of SIRT1 on NMDA-induced excitotoxicity were attributed to its deacetylase activity; and third, inhibition of p53 acetylation might be one of the mechanisms underlying SIRT1-mediated neuroprotection.

In this study, we found that either preincubation of cortical neurons with RSV or overexpression of WT-SIRT1 in the SH-SY5Y cell line prevented NMDA-induced excitotoxicity including a decrease in cell viability, an increase in LDH release, and an increase in cell death, suggesting that SIRT1 has neuroprotection in NMDA-induced excitotoxicity. As has been reported, activation of SIRT1 using RSV has protection against disorders of the nervous system, for example, brain ischemia reperfusion injury [11], Alzheimer's disease, Parkinson's disease [12], and traumatic CNS injury [13]. We also found that Sirtinol, a pharmacological inhibitor of SIRT1, abolished the protection of RSV against NMDA-mediated nerve injury, indicating that the neuroprotective role of RSV is possibly achieved by activation of SIRT1. It has been shown that RSV ameliorates motor neuron degeneration and improves survival mainly through increasing the expression of SIRT1 in the SOD1^G93A^ mouse model of amyotrophic lateral sclerosis [14]. Inhibition of SIRT1 increased axonal injury and activation of SIRT1 prevented neuronal insults in *in vivo* and *in vitro* models of Wallerian degeneration [15, 16]. Further evidence demonstrates that SIRT1 overexpression can also play a protective role in a variety of *in vivo* and *in vitro* models of nerve injury. Overexpression of SIRT1 improves motor function, reduces brain atrophy, and attenuates mutant-HTT-mediated metabolic abnormalities in a mouse model of Huntington's disease [17]. Overexpression of SIRT1 protein in neurons protects against experimental autoimmune encephalomyelitis through activation of multiple SIRT1 targets [18]. In addition to the findings in support of the protective effects of SIRT1 on neurodegeneration, there are also contradictory studies reporting the opposite effect. In this respect, it was shown that SIRT1 inhibition reduces IGF-1/IRS-2/Ras/ERK1/2 signalling and protects neurons [19].

Further observation shows that RSV significantly ameliorated NMDA-reduced SIRT1 deacetylase activity in primary neurons, and this amelioration was prevented when SIRT1 activity was inhibited by Sirtinol. Therefore, it raises the possibility that the deacetylase activity is required for SIRT1's neuroprotection in the excitotoxicity model. In addition, we observed that overexpression of WT-SIRT1 reversed NMDA-induced reduction of SIRT1 mRNA, SIRT1 protein level, and SIRT1 deacetylase activity and inhibition of NMDA-induced insults of SH-SY5Y cell. However, overexpression of DN-SIRT1 increased the levels of SIRT1 mRNA and protein reduced by NMDA but had no effect on NMDA-induced decrease in the deacetylase activity and also did not inhibit subsequent excitotoxic cell death. These results clearly indicated that SIRT1 deacetylase activity is crucial to the neuroprotective effects of SIRT1 in NMDA-induced insults. A previous work by a number of other laboratories has also established that RSV potentiates SIRT1 activity and provides neuroprotection in recurrent stroke models [20], stress resistance, and prosurvival effects [21]. The deacetylase-deficient SIRT1 (H363Y) completely eliminated the protective effects of SIRT1 in HD models [17]. Modulation of sirtuin activity has been shown to impact the course of several aggregate-forming neurodegenerative disorders including Alzheimer's disease, Parkinson's disease, Huntington's disease, amyotrophic lateral sclerosis, and spinal and bulbar muscular atrophy [22]. The above evidences and our results support that SIRT1 deacetylase activity is critical to its neuroprotection. But there are different opinions about SIRT1 on neuronal survival that SIRT1-mediated neuroprotection is independent of its deacetylase activity, and this mechanism might involve interactions between SIRT1 and other apoptosis-regulatory proteins [23].

Additionally, we found that overexpression of WT-SIRT1 significantly inhibited NMDA-induced p53 acetylation and subsequent neurotoxicity. However, DN-SIRT1 overexpression has no such effect. The findings suggest that SIRT1 might provide potent neuroprotection against NMDA insult through regulating p53 acetylation. As a deacetylase, SIRT1 is known to deacetylate and modulate the activity of key transcription factors, such as P53, NF-*κ*B, PGC-1*α*, LKB1, TSC2, HSF1, and other substrates, which participate in the adjustment of the process of a variety of injuries. The available evidence indicated that SIRT1 reduces the activity of p53 by removing these acetyl groups that inhibits apoptosis and promotes cell survival [24, 25]. In this experiment, we observed that NMDA induced p53 acetylation which may be one of the mechanisms of inducing neuronal death via apoptosis. Acetylation is thought to be a key event for p53 activation and Ace-p53 induces apoptosis and is involved in neuronal death [26, 27]. Together, these experiments demonstrate that deacetylation of p53 is at least in part required for SIRT1-mediated neuroprotection in the excitotoxicity model.

SIRT1 is an endogenous neuroprotective factor and mediates protection through different pathways. The mechanisms of the neurotoxic effects of NMDA are very complex including calcium overload, oxidative stress, mitochondrial dysfunction, cell necrosis, and apoptosis [28]. Nonetheless, our results suggest that NMDA may inhibit the activity of SIRT1 and weakens the protective effect of SIRT1. Subsequent experimental observation confirmed this speculation, because SIRT1 activation by RSV or overexpression of SIRT1 ameliorates NMDA-induced neurotoxicity and exerts the neuroprotection.

In summary, a growing body of evidence has confirmed the neuroprotective effects of SIRT1. The finding of the present study suggests that SIRT1 might be a therapeutic target for certain neurological diseases related to NMDA-mediated excitotoxicity.

## Abbreviations

SIRT1: Silent information regulator 1
RSV: Resveratrol
LDH: Lactate dehydrogenase
FBS: Fetal bovine serum
PI: Propidium iodide
Ace-p53: Acetylated p53
AD: Alzheimer's disease
HD: Huntington's disease.
